# Cultivating a community of practice: the evolution of a health information specialists program for public librarians

**DOI:** 10.5195/jmla.2017.83

**Published:** 2017-07-01

**Authors:** Shari Clifton, Phill Jo, Jean Marie Longo, Tara Malone

## Abstract

**Background:**

To help improve the culture of health in Oklahoma—a state that frequently ranks poorly on multiple measures of health and wellness—faculty librarians from an academic health sciences library sought to create a collaborative network of health information professionals in Oklahoma’s public libraries through the implementation of the Health Information Specialists Program.

**Case Presentation:**

Health sciences librarians offered a variety of consumer health information courses for public library staff across the state of Oklahoma for three years. Courses were approved by the Medical Library Association for credit toward the Consumer Health Information Specialization. A total of seventy-two participants from public libraries attended the courses, sixty-five achieved a Level I Consumer Health Information Specialization, and nine went on to achieve Level II.

**Conclusions:**

Feedback from participants in the Health Information Specialists Program has indicated a positive impact on the health information expertise of participants, who in turn have used the knowledge that they gained to help their patrons.

## BACKGROUND

Oklahoma is among the unhealthiest states in the nation and has been consistently ranked as one of the ten worst performers on a variety of health and wellness measures since 2000 [1]. The most recent *State of the State’s Health Report* highlights a number of Oklahoma’s challenges including high death rates due to cancer, heart disease, stroke, and unintentional injury; significant prevalence of smoking, diabetes, and obesity; and low adoption of healthy behaviors such as consumption of fruits and vegetables and physical activity [2]. In an effort to contribute to solutions for these problems, faculty members from the Robert M. Bird Library (BHSL) at the University of Oklahoma Health Sciences Center (OUHSC) partnered with public library staff to expand consumer health information outreach throughout the state.

The BHSL has a long history of active engagement with public libraries and health information outreach efforts across Oklahoma. Previous activities include classes for staff in public and school libraries, direct-to-consumer outreach for public library patrons, exhibits and presentations at professional meetings, and assistance with library programs, health fairs, and community events focused on health and wellness. Serving seven colleges, the BHSL provides resources and services to OUHSC students, faculty, and staff who work in a wide array of teaching, research, and clinical domains. In addition, as home to the largest health collection in the state, the BHSL is open to the public and is a designated Resource Library in the National Network of Libraries of Medicine, South Central Region (NNLM SCR).

Because public libraries in Oklahoma are embedded in their communities and have a tradition of meeting the needs of diverse user populations, BHSL faculty view public libraries as crucial players in connecting individuals with the health information they need and improving Oklahoma’s culture of health. To engage public library staff in an exploration of the relationships between information access and health, BHSL faculty set out to establish a community of practice. Wenger et al. define a community of practice as “a group of people who share a concern, a set of problems, or a passion about a topic, and who deepen their knowledge and expertise by interacting on an ongoing basis” [3].

In 2013, BHSL faculty incorporated this premise into the development and evolution of the Health Information Specialists Program with goals to:

- establish a network of health information professionals in Oklahoma
- contribute to the health and wellness of the communities they serve
- expand knowledge of and access to reliable health information
- solidify partnerships for the delivery of quality health information services
- nurture collaboration between the BHSL and public libraries throughout the state

A cornerstone of Oklahoma’s Health Information Specialists Program has been the opportunity for participants to achieve the Medical Library Association’s (MLA’s) Consumer Health Information Specialization (CHIS), a continuing education initiative designed to increase knowledge and expertise in the consumer health field. There are two CHIS levels:

- Level I recipients must complete twelve hours of approved courses or activities, with four hours of coursework focused on the administration and management of consumer health services and a minimum of eight hours of coursework from the MLA-approved CHIS course list
- Level II recipients must complete twenty-four hours of approved courses or activities, with four hours focused on the administration and management of consumer health services and eighteen hours of coursework from the MLA-approved CHIS course list [4]

## STUDY PURPOSE

Oklahoma’s Health Information Specialists Program was designed to provide continuing education and networking opportunities for public library staff who are interested in consumer health resources and services. From its inception, a key component of the program has included funds to cover the CHIS fee for all participants who complete the required number of courses.

## CASE PRESENTATION

### Pilot project (2013–2014)

In 2012, BHSL faculty were asked to provide consumer health information training for staff members in the Metropolitan Library System (MLS). System offices for the MLS are located in Oklahoma City, with nineteen libraries serving diverse communities throughout Oklahoma County. Participants evaluated the training session favorably, and as a result, staff members at both the MLS and the BHSL pursued further collaborations to expand health information outreach in Oklahoma County. Following a meeting in January 2013 and additional discussions, the plan to build a cohort of MLS staff with consumer health expertise was formulated, and the Health Information Specialists Program was conceived. Funding for the pilot project was obtained through an outreach award from the NNLM SCR.

Pilot project participants were recruited by MLS staff, and all courses were held in a computer lab at the MLS headquarters. Before courses commenced, participants and program coordinators from MLS and the BHSL held conference calls to introduce the program and gather feedback about course topics that participants were interested in. After a preliminary list was developed, 18 participants ranked 11 topics in an online survey compiled by BHSL faculty. Course offerings for both the pilot project and phase 2 were prioritized by selecting the courses that appeared most frequently in the top 5 choices of respondents. The most popular topics were health information resources and services in Oklahoma (83%); mental health information resources (83%); health information resources for older adults (78%); nutrition information resources (67%); drug information resources (56%); resources for kindergarten through twelfth grade (K–12) educators, parents, children, and teens (39%); and cancer information resources (33%).

Seventeen public library staff participated in the pilot project (Table 1). Five unique hands-on courses were offered for the pilot project (Table 2). Two courses, “Food for Thought” and “Caring for the Mind,” were taught by NNLM SCR staff; BHSL faculty served as instructors for the other courses. One course, “Just What the Doctor Ordered,” was offered twice to allow participants with potential conflicts to complete the course, as this class fulfilled the CHIS requirement of four hours of coursework focusing on the administration and management of consumer health services. Each course was evaluated using MLA’s standard evaluation form, and summary evaluation data are provided in Table 3 and Table 4.

**Table 1:** Health Information Specialists Program: participation

	Pilot project (2013–2014)	Phase 2 (2014–2015)	Phase 3: expansion (2015–2016)
Participants	17	16	47
Participants achieving Consumer Health Information Specialization (CHIS) Level I	17	7	41
Participants achieving CHIS Level II		9	
Participating libraries	13	13	30

**Table 2:** Health Information Specialists Program: course summaries

Courses	Pilot project (2013–2014)	Phase 2 (2014–2015)	Phase 3: expansion (2015–2016)
Cancer Information Inside and Out (3 contact hours)		♦	
Caring for the Mind: Providing Mental Health Information at Your Library (3 contact hours)	♦		
Close to Home: Health Information Resources and Services for Oklahomans* (3 contact hours)	♦	♦	♦
Drug Information Resources for Consumers (3 contact hours)	♦	♦	♦
Food for Thought: Exploring Nutrition Resources (3 contact hours)	♦		
Health Information for Special Populations† (3 contact hours) (American Indians, veterans, LGBTQ, non-English speaking)		♦	
Healthy Aging at Your Library: Connecting Older Adults to Health Information (3 contact hours)		♦	♦
Just What the Doctor Ordered: Consumer Health Information @ Your Library (4 contact hours)	♦	♦	♦
K–12 Health Information Resources† (3 contact hours)		♦	
Promoting Health Literacy Through Easy-to-Read Materials (3 contact hours)		♦	
Will Duct Tape Cure My Warts? Examining Complementary and Alternative Medicine (3 contact hours)		♦	♦

* Due to its focus on Oklahoma, this course is not available in the Medical Library Association’s (MLA’s) MEDLIB-ED. Participants can submit an Individual Participant Request (Form IPR) to apply this course to the CHIS.

† These courses are no longer available via MLA’s MEDLIB-ED.

**Table 3:** Health Information Specialists Program: course evaluation summaries*

	Pilot project (2013–2014)		Phase 2 (2014–2015)		Phase 3: expansion (2015–2016)	
Sessions taught	6*		10		25	
Evaluations distributed	58		77		206	
Evaluations returned	57		77		204	
Participant ratings						
I acquired knowledge and skills I can use	Agree	(96%)	Agree	(95%)	Agree	(96%)
	Somewhat agree	(4%)	Somewhat agree	(5%)	Somewhat agree	(4%)
Session objectives met my expectations	Agree	(86%)	Agree	(94%)	Agree	(92%)
	Somewhat agree	(14%)	Somewhat agree	(5%)	Somewhat agree	(8%)
	No response	(1%)				
Instructional materials were relevant/useful	Agree	(88%)	Agree	(94%)	Agree	(92%)
	Somewhat agree	(11%)	Somewhat agree	(2%)	Somewhat agree	(6%)
	Somewhat disagree	(1%)	No response	(4%)	No response	(2%)
Session content was well organized	Agree	(88%)	Agree	(97%)	Agree	(94%)
	Somewhat agree	(12%)	Somewhat agree	(3%)	Somewhat agree	(6%)

* Evaluations were not available for the Food for Thought course during the pilot project.

**Table 4:** Health Information Specialists Program: selected course evaluation comments

	Pilot project (2013–2014)	Phase 2 (2014–2015)	Phase 3: expansion (2015–2016)
What part of this session was most helpful?	Handouts are great—also the exercises were very practical and good ‘practice’ for real reference scenarios.	Sharing information; working as a team; exercises; resource materials.	Vetted, yet robust list of database resources that were toured and then we got to practice.
	The time given to find answers on our own; exploring the various resources.	The resources provided were wonderful! I thought of about ten good programming ideas from this class alone.	Scavenger hunt/independent practice.
	The discussion of health literacy and trying to see things from the point of view of the customer was very valuable.	I always appreciate learning about new resources I may not have encountered before. These can definitely be useful when there’s a gap in my library’s collection.	Wonderful resources! I can feel confident I am giving our patrons accurate information.
	Lots of great resources; I was unfamiliar with the Oklahoma-specific ones.	Hands-on exercises were helpful and taught skills I will use.	Hands-on learning and resources to explore.
	Excellent class! I will use all of the resources!	Applying what was learned through meaningful exercises.	The folder to take home with me so I can review and reuse what we went over.
	Having the opportunity to test these websites; to get a good feel for how they may be used.		The information and class sharing of ideas and programs.
			I really like that we get to navigate the sites and look up info.
Additional comments	I was so glad there wasn’t a group work component! I’m really sick of that!	I really enjoyed this class and feel like I learned about some resources I’ve never used.	Collection development not really relevant to me but nice to go over.
	If this series is offered again in the future it would be great if it was a weekly class, rather than monthly.	I’m a fan of group work!	Very useful, great content. I was able to be more familiar with the helpful websites. AWESOME class and info.
	I wish the class had been longer for more in-depth exploration of resources!	I really enjoyed this series and look forward to taking these tools back to my library and using them to help our customers.	Can the database links (resource sheet) come with brief audience/summaries? Good to hand out to interested parties. Great presentation!
	I thought this was a really useful overview course and look forward to attending more!	Everything in this session was helpful and the information gained is applicable to my job.	

### Phase 2 (2014–2015)

After the pilot project, two conference calls with project participants were held, during which a proposal to expand the program to other MLS staff members received unanimous support. In addition, participants requested new courses that would allow them to continue to develop their consumer health expertise. MLS coordinators also expressed their commitment to continue the program and to recruit new participants for phase 2. As with the pilot project, phase 2 of the Health Information Specialists Program was supported with funds from the NNLM SCR.

Nine unique courses were selected for phase 2; five courses addressed areas new to the program curriculum (Table 2). Again, all courses were taught in a computer lab at the system headquarters. Eight of the original participants returned to complete their Level II specialization, and eight new participants joined the program. One of the new participants completed twenty-four hours of coursework to achieve CHIS Level II (Table 1). Each course in phase 2 was taught by BHSL faculty and was evaluated using MLA’s standard evaluation form, and summary evaluation data are provided in Table 3 and Table 4.

### Phase 3: Expansion (2015–2016)

Based on positive feedback from participants in the initial two years of the project, BHSL faculty decided to expand program participation beyond the partnership with MLS. Key stakeholders from the Oklahoma Public Library Directors’ Council as well as the public library consultants at the Oklahoma Department of Libraries were consulted to discuss strategies for course delivery, participant recruitment, training site selection, and marketing. Partial funding for phase 3 of the Health Information Specialists Program was provided by the NNLM SCR; due to the volume of participation, supplementary funds from the BHSL were also required.

A series of five courses was offered in phase 3; courses were selected by BHSL faculty based on course evaluation data from the first two years of the program (Table 1). Each series was taught at five regional training sites across the state of Oklahoma: Altus Public Library (Southern Prairie Library System) in the southwest, Ardmore Public Library in the south-central corridor, Enid Public Library in the northwest, Norman Central Library (Pioneer Library System) centrally located just south of the greater Oklahoma City metropolitan area, and Hardesty Regional Library (Tulsa City-County Library System) in the northeast [5]. The inclusion of multiple training sites reflected the geographic diversity of Oklahoma, ensured that training would not place an undue travel burden on participants, and allowed participants the option to travel to another site to make up missed courses in order to achieve their specializations. BHSL faculty taught all phase 3 courses, and data from MLA’s standard evaluation form are provided in Table 3 and Table 4.

In addition to CHIS-approved coursework, participants in phase 3 earned continuing education units (CEUs) applicable to the Public Librarian Certification Program administered by the Oklahoma Department of Libraries and designed to develop professional standards for Oklahoma’s public libraries [6]. Adding this element was important because many public libraries in the state require that their staff obtain this certification.

To gather feedback from participants and to inform the program developers’ next steps, a 15-question online survey was distributed in spring 2016 (supplemental appendix). The survey link was emailed to 72 individuals who participated in the first 3 years of the Health Information Specialists Program, with a response rate of 50%. All respondents agreed or strongly agreed that taking part in the program improved their knowledge and skills in locating health information for their communities or for themselves. One participant said that the program “provides a bridge to the tools and resources needed for health information literacy.”

Other respondents to the survey discussed the increased confidence that they felt after participating in the program. One indicated that the program “provided me with the skills necessary for a health reference interview,” while another shared, “I have helped several patrons since the courses and have found them useful because I was able to do more than just say, ‘Let’s Google it and see if we find something.’” Another theme emerging from the survey was appreciation for the networking and active learning opportunities that the in-person courses provided. The program also helped participants develop new health-related programs in their own libraries. For example, one respondent shared, “As a public librarian, I have used the information [from the program] to better serve library customers. I have incorporated the information into pathfinders for customers and shared the information with coworkers. Our library now has a monthly program focused on health information.”

Of the survey respondents, 78% indicated that they participated in the program to learn more about health information resources and services, followed by 48% who were interested in completing the CHIS and 30% who needed CEUs applicable to the Public Librarian Certification. When asked about delivery methods for future courses, 67% of respondents preferred in-person classes; however, there was also interest in utilizing online platforms (13%), videoconferencing (10%), or a combination of methods (26%). Topics of interest to address or revisit in the future included nutrition (72%), health literacy (62%), mobile apps (56%), K–12 health information (51%), and cancer information (46%).

When asked to share suggestions that would improve the program, the most common theme that respondents expressed was related to course length and/or structure. Responses included lengthening the courses to explore topics and resources more thoroughly, grouping multiple classes on the same date, and providing alternatives to in-person instruction. A few respondents commented that the courses would be stronger with additional interactive components and more continuity between courses.

BHSL faculty deliberately addressed these suggestions in the plan for phase 4. Based on responses to the cumulative survey, individual course evaluations, and informal participant feedback, a proposal was prepared and submitted to the NNLM SCR outlining phase 4 of the Health Information Specialists Program. In 2016, funding was approved, and phase 4 activities commenced. To introduce variety and to appeal to both new and returning participants, phase 4 will include a consumer health continuing education day featuring three MLA webinars that are relevant to consumer health; the pilot of an online, asynchronous course focused on mobile apps; five book discussions; fifteen in-person classes at three training sites; and three additional in-person courses focused on grant writing and program evaluation offered over a period of two days.

## CONCLUSIONS

The National Library of Medicine (NLM) and many health sciences librarians recognize the importance of building and maintaining relationships with public library staff to facilitate the delivery of consumer health information. Wood and colleagues report on a pilot project initiated by NLM in 1998 with public libraries around the United States; while some of the resource references are dated, the ideas for engaging public libraries in consumer health information delivery continue to resonate [7]. A more recent article describes a variety of collaborations between health sciences and public libraries, including train-the-trainer projects and activities focused on public library patrons [8]. Also, Zionts et al. report on their project in Pittsburgh that, like Oklahoma’s Health Information Specialists Program, incorporates the CHIS for public library staff who participate in their Health Information Fellowship initiative [9]. A common theme throughout these projects is the need to engage public library staff in developing and implementing programming, to adapt existing programs or services to their needs and the needs of their patrons, and to evaluate and disseminate these types of projects through presentations and publications. Keeping these elements at the forefront of planning and activities has been the key lesson learned from the evolution of this program.

In Oklahoma, the challenges to optimal health are many. The BHSL also faces numerous demands, including acquiring funding, addressing the information needs of diverse user groups, and carving out faculty time for the development of outreach proposals, projects, and activities. In spite of these difficulties, BHSL faculty are committed to ensuring that the Health Information Specialists Program continues to evolve to meet the diverse needs of Oklahoma’s public library staff and their patrons. The development of a true community of practice remains a core value of the program and is contingent upon the quality of the program and the ability to offer varied opportunities for education, collaboration, and networking to current and previous participants on a regular basis. While identifying an ongoing funding source for the CHIS participant fee is the biggest challenge to program sustainability, BHSL faculty are committed to protecting the program investments made to date and ensuring that new activities and collaborations are available in the future.

## SUPPLEMENTAL FILE

Appendix Health Information Specialists Program, 2013–2016, cumulative survey questions
