# Supplementary material for: Cultivating a community of practice: the evolution of a health information specialists program for public librarians
Source: J Med Libr Assoc. 2017 Jul 1;105(3):254–61. doi: 10.5195/jmla.2017.83 (PMC5490704; doi:10.5195/jmla.2017.83)
Supplement: Appendix [file jmla-105-254-a001.pdf]

Journal of the Medical Library Association

Supplemental content to
J Med Libr Assoc. Jul;105(3): dx.doi.org/10.5195/jmla.2017.83
www.jmla.mlanet.org
© Clifton, Jo, Longo, Malone 2017

Cultivating a community of practice: the evolution of a health information specialists program for public librarians

Shari Clifton, AHIP; Phill Jo; Jean Marie Longo; Tara Malone

APPENDIX

Health Information Specialists Program, 2013–2016, cumulative survey questions

Question 1: When did you participate in the Health Information Specialists Program? (Select all that apply if you attended classes in more than one year)
☐ 2013–2014
☐ 2014–2015
☐ 2015–2016

Question 2: If you completed the required number of classes for your Consumer Health Information Specialization (CHIS) from the Medical Library Association (MLA), what level did you achieve?
☐ Level I
☐ Level II
☐ Did not complete
☐ Not sure

Question 3: Are you interested in future opportunities so that you can achieve the CHIS, move to Level II, or gain additional continuing education credits?
☐ Yes
☐ Maybe
☐ No

Question 4: Why did you participate in the Health Information Specialists Program? (Select all that apply)
☐ I was told to by my supervisor/manager
☐ I was interested in obtaining continuing education units (CEUs) that could be applied to the Public Librarian Certification Program administered by the Oklahoma Department of Libraries
☐ I was interested in obtaining the CHIS
☐ I wanted to learn more about health information resources and services
☐ Other

Question 5: What is your preferred delivery method for training? (Check all that apply.)
☐ In-person classes
☐ Online sessions via meeting software such as GoToMeeting or WebEx
☐ Videoconferencing
☐ A combination of the methods mentioned above
☐ Other
☐ No preference

Question 6: What is your preferred location for training? Drag and drop your choices into your preferred order.
☐ My office or library
☐ A regional location you can reach within 1–2 hours
☐ A central location such as the Oklahoma Department of Libraries
☐ Other
☐ No preference

Question 7: What is your preferred class length?
☐ Full day (6–7 hours)
☐ Half day (3–4 hours)
☐ Brief sessions (1–2 hours)
☐ Varied lengths depending on the course content
☐ Other
☐ No preference

Question 8: If you have a training location we should investigate, please indicate the site and contact person below.

Question 9: Which of the topics below are you interested in for future classes? (Check all that apply)
☐ Cancer information
☐ Health information resources for K–12 students, teachers, and parents
☐ Health information for special populations (American Indians/veterans/LGBT/non-English speakers)
☐ Nutrition information resources
☐ Health literacy
☐ Mobile apps
☐ Other

Question 10: Are you interested in collaborating with librarians from the Bird Health Sciences Library to provide health information at your library or in your community?
☐ Yes
☐ No
☐ I'm interested but do not have any ideas right now
☐ If yes, do you have any ideas/suggestions at this time:

Question 11: Based on my participation in the Health Information Specialists Program, I would be interested in further training opportunities taught and/or coordinated by librarians at the Bird Health Sciences Library.
☐ Strongly agree
☐ Agree
☐ Neither agree or disagree
☐ Disagree
☐ Strongly disagree

Question 12: My participation in the Health Information Specialists Program has improved my knowledge and skills in locating health information for my community and/or myself.
☐ Strongly agree
☐ Agree
☐ Neither agree or disagree
☐ Disagree
☐ Strongly disagree

Question 13: What did you like most about the Health Information Specialists Program?

JMLA Journal of the Medical Library Association

SC

Supplemental content to
J Med Libr Assoc. Jul;105(3): dx.doi.org/10.5195/jmla.2017.83
www.jmla.mlanet.org
© Clifton, Jo, Longo, Malone 2017

Question 14: What suggestions do you have for improvements in the Health Information Specialists Program?

Question 15: Stories are powerful, and we are very interested in hearing about your experiences. Please share/describe any ways you have utilized the knowledge, resources, or relationships you gained through the Health Information Specialists Program to serve your patrons/customers, colleagues, family members, etc.
